# Toll-like receptor 4 inhibition prevents autoimmune diabetes in NOD mice

**DOI:** 10.1038/s41598-019-55521-z

**Published:** 2019-12-18

**Authors:** Mohamed Alibashe-Ahmed, Estelle Brioudes, Walter Reith, Domenico Bosco, Thierry Berney

**Affiliations:** 1 Cell Isolation and Transplantation Center, Diabetes Center, University of Geneva Hospitals, Geneva, Switzerland; 2 Department of Pathology and Immunology, University of Geneva, 1211 Geneva 4, Geneva, Switzerland

**Keywords:** Autoimmunity, Endocrinology

## Abstract

TLR4 is a transmembrane receptor of the innate immune system that recognizes LPS from gram-negative bacteria. Its stimulation induces pro-inflammatory responses and modulates adaptive immunity. Our aim is to determine the role of TLR4 in the activation and proliferation of T lymphocytes in the onset of autoimmune diabetes, using the non-obese diabetic (NOD) mouse model. Antigen-specific activation and proliferation of diabetogenic T cells were assessed *in vitro* by Carboxyfluorescein succinimidyl ester (CFSE) dilution, in presence of vehicle or CLI-095, a cyclohexene derivative that inhibits TLR4 signaling. NOD mice were treated with vehicle or CLI-095 and sacrificed either before or after the onset of autoimmune diabetes. T lymphocyte activation and proliferation were evaluated in treated and control mice. Insulitis was analyzed by histology and diabetes incidence was determined in treated and control mice. Our results demonstrate that TLR4 blockade decreases CD4+ T lymphocyte activation and auto-antigen-specific proliferation both *in vitro* and *in vivo*, decreases the infiltrative insulitis and finally prevents the onset of spontaneous diabetes. Taken together, our data demonstrate that TLR4 signaling contributes to the development and maintenance of autoimmune diabetes. The immunomodulatory effect of CLI-095 could be part of a preventive strategy targeting patients at risk for type 1 diabetes.

## Introduction

Type 1 diabetes (T1D) is a multifactorial, inflammatory and autoimmune disease. The importance of genetic and immunological factors in T1D is highlighted by the activation of T lymphocytes by autoantigens. Recently, new evidences have demonstrated a role of innate receptors in the development of autoimmunity^1,2^. Pathogen-recognition receptors in diabetes have been widely studied, but little is known about the role of TLR4 in the pathogenesis of this disease. It has been suggested that alteration of gut immunity and exposition to certain pathogens might be important contributors to the pathogenesis of T1D. In this context, TLR4 could be a major player in diabetes pathogenesis, since it recognizes bacterial LPS but also damage associated molecular patterns (DAMPs). It has been shown that insulitis and the autoimmune process leading to overt T1D involve TLR4-expressing cells of the innate and adaptive immune system, but contradictory results have been reported on the development of T1D in mouse models bearing the general knock-out of TLR4^3,4^. In addition, clinical data have shown not only increased levels of TLR4 ligands in diabetic patients, but also increased levels of TLR4 expression in their innate immune cells^5,6^.

CLI-095 (also known as TAK-242), is a selective inhibitor of TLR4 that targets the intracellular domain of TLR4 and has been demonstrated to be highly effective at blocking human and murine TLR4 signalling^7,8^. NOD mice, share genetic and immunological similarities with human T1D^9^. They produce autoantibodies, develop insulitis and progress to spontaneous autoimmune diabetes. In this work, we investigated the implication of the activation of TLR4 on diabetes onset by using CLI-095 in the NOD mouse model.

Our working hypothesis is that TLR4 may be involved in the pathogenesis of diabetes and that its blockade may prevent or delay the onset of autoimmunity. More specifically, we assessed *in vivo* and *in vitro*, the impact of TLR4 blockade on the function of diabetogenic T lymphocytes, and evaluated its impact on insulitis and the development of diabetes.

## Results

### TLR4 blockade impairs TCR/CD3-mediated activation of CD4+ T lymphocyte and their antigen-specific proliferation

**Figure 1:**
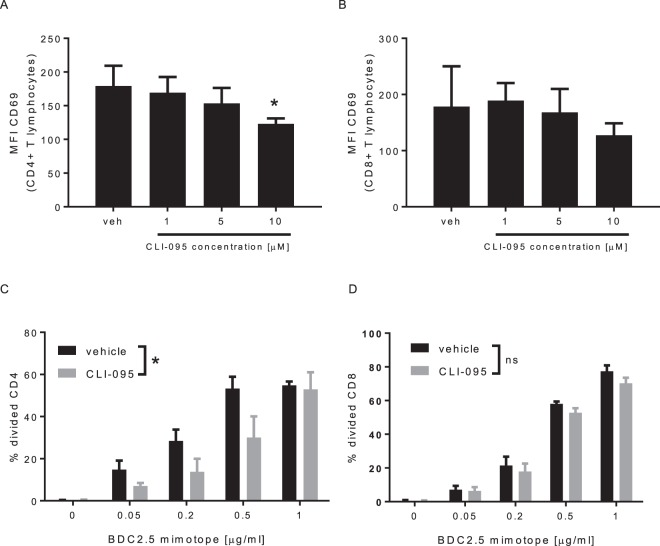
CLI-095 inhibits CD4+ T lymphocytes activation and antigen-specific proliferation *in vitro*. (**A,B**) Expression of CD69, after the activation of CD4+ and CD8+ T cells with anti-CD3 antibody, in presence of CLI-095. (**C,D**) Effect of CLI-095 or vehicle on BDC2.5 CD4+ and CD8+ T lymphocytes antigen-specific activation. Data are means ± SEM from three (**A,B**) and five experiments (**C,D**) *p ≤ 0.05.

To evaluate the impact of TLR4 on the TCR/CD3-mediated activation, CD4+ and CD8+ lymphocytes were activated with anti-CD3 antibody in absence (vehicle) or presence of CLI-095 (Fig. 1). We observed that CLI-095 decreases in a dose dependent manner the activation of CD4+ T lymphocytes (Fig. 1A). No significant effect was observed on CD8+ T lymphocytes (Fig. 1B). We then evaluated the effect of CLI-095 on antigen-specific proliferation of T lymphocytes. To this end, BDC2.5 CD4+ and CD8+ T lymphocytes were activated, with increasing concentrations of a peptide that mimics a β-cell antigen recognized by the BDC2.5 lymphocytes (BDC2.5 mimotope), in absence (vehicle) or presence of CLI-095, as compared to control (Fig. 1C,D). Proliferation was analysed by CFSE dilution. CLI-095 decreased the proliferation of CD4+ T lymphocytes (Fig. 1C). No effect of CLI-095 was observed on the antigen-specific proliferation of CD8+ T lymphocytes (Fig. 1D). In summary, these data show that TLR4 blockade specifically inhibits the activation of CD4+ T lymphocytes and prevents their antigen-specific proliferation.

### TLR4 inhibition prevents the activation of diabetogenic CD4+ T lymphocytes *in vivo*

**Figure 2:**
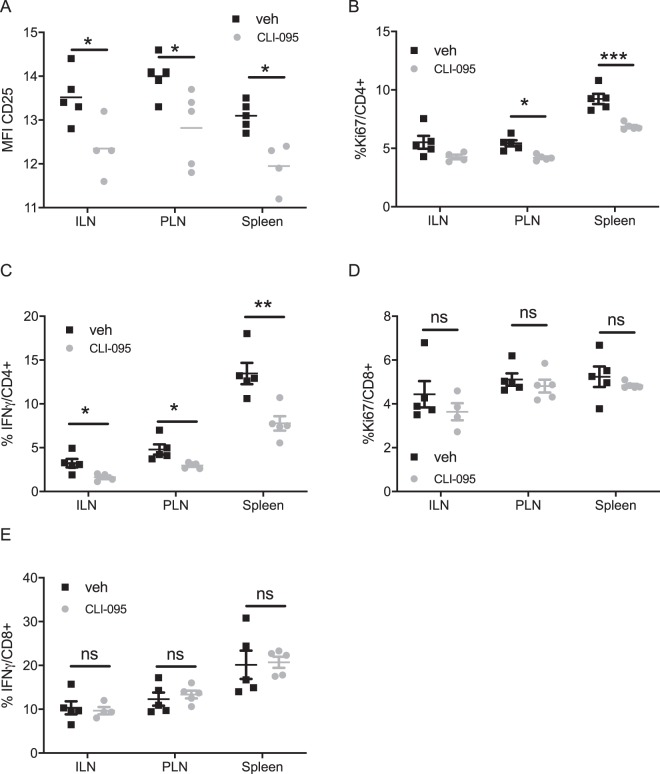
Effect of TLR4 blockade on T lymphocytes in NOD mice after treatment with CLI-095: (**A**) Expression of CD25 on CD4+ T lymphocytes from the pancreatic lymph nodes (PLN), inguinal lymph node (ILN) and from the spleen of the treated mice (grey) and the controls (black). (**B**) %Ki67+CD4+ T lymphocytes in the different lymphoid organs. (**C**) %IFNγ+CD4+ T lymphocytes in the different lymphoid organs. (**D**) %Ki67+CD8+ T lymphocytes in different lymphoid organs. (**E**) %IFNγ+CD8+ T lymphocytes in different lymphoid organs. Data are means ± SEM collected from five mice *p ≤ 0.05, **p ≤ 0.01, ***p ≤ 0.001

We then evaluated the effect of CLI-095 on the activation of the diabetogenic T lymphocytes, prior the onset of autoimmune diabetes in NOD mice (Fig. 2). We analysed T lymphocyte activation in the local pancreatic lymph nodes (PLN), in the spleen and in the distant inguinal lymph nodes (ILN) of 12-week old NOD mice treated with vehicle or CLI-095. Compared to treatment with vehicle, treatment with CLI-095 significantly decreased activation and proliferation of the CD4+ T lymphocytes, measured by expression of CD25 and Ki-76 respectively, both in PLN and spleens (Fig. 2A,B). Compared to vehicle, CLI-095 also decreased the number of IFNγ-producing CD4+ lymphocytes in the ILN, PLN and spleens (Fig. 2C). However, no effect of CLI-095 was observed on the activation, the proliferation and the number of IFNγ-producing CD8+ T lymphocytes (Fig. 2D,E). In accordance with our *in vitro* results, these data indicate that TLR4 blockade specifically inhibits the activation of CD4+ T lymphocytes in NOD mice.

### TLR4 inhibition prevents the activation of autoreactive CD4+ T lymphocytes

**Figure 3:**
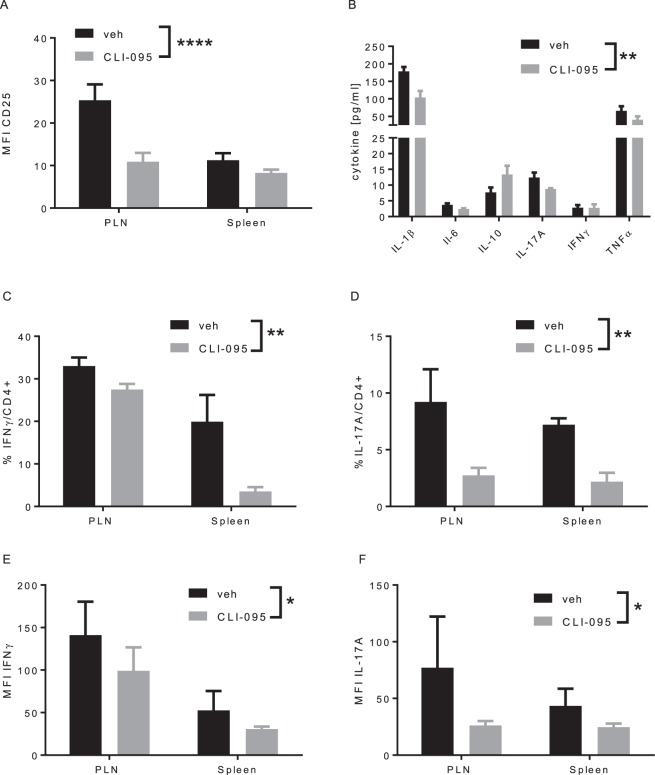
Effect of TLR4 blockade on CD4+ T lymphocytes in model of adoptive transfer of diabetes. (**A**) expression of CD25 on BDC2.5 CD4+ T cells in pancreatic lymph nodes (PLN) and spleen. (**B**) Cytokine secretion measured in sera of treated and control animals. (**C,D**) %IFNγ- and IL-17A-producing BDC2.5 CD4+ T cells in PLN and spleen. (**E,F**) quantification of the production IFNγ of IL-17A in PLN and spleen. Data are means ± SEM collected from four mice *p ≤ 0.05, **p ≤ 0.01, ***p ≤ 0.001

To further investigate the impact of TLR4 on autoreactive CD4+ T lymphocytes, we performed an adoptive transfer of naïve BDC2.5 CD4+ T lymphocytes to immunocompromised NOD/*Rag1*^*−/−*^ mice, treated with CLI-095 or vehicle (Fig. 3). In this model of diabetes transfer, CLI-095 decreased the activation of CD4+ T lymphocytes and the secretion of pro-inflammatory cytokines (Fig. 3A,B). In this regard, the percentage of IFNγ-and IL-17A- producing CD4+ T lymphocytes in the PLN and spleen was lower in CLI-095-treated mice compared to vehicle-treated mice (Fig. 3C,D). In addition, CLI-095 decreased the content of pro-inflammatory cytokines in lymphocytes from CLI-095-treated mice (Fig. 3E,F). These data show that, in this model of type 1 diabetes, TLR4 blockade impairs the activation of CD4+ T lymphocytes and the production and secretion of pro-inflammatory cytokines, as observed in NOD mice.

### TLR4 inhibition prevents infiltrative insulitis in NOD mice

**Figure 4:**
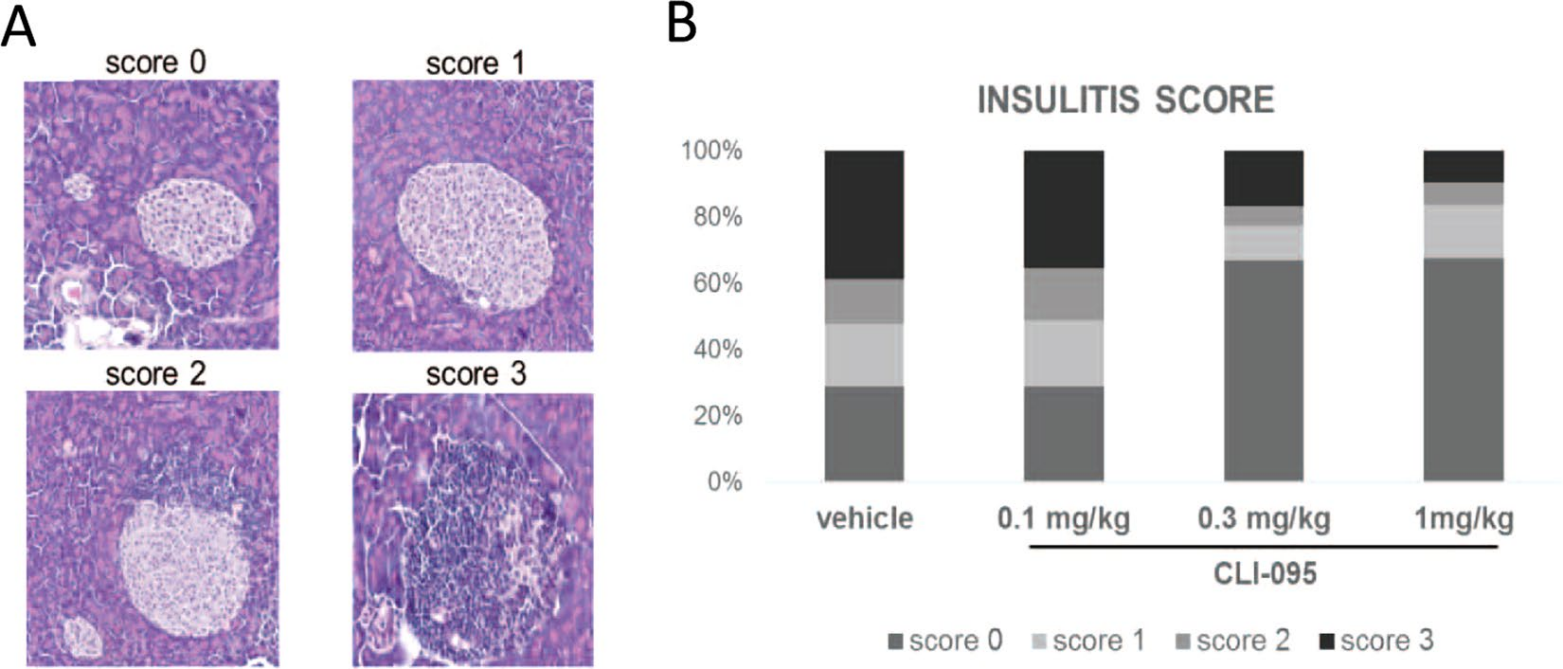
Insulitis score in 12-week female NOD mice. (**A**) Representative histological images of insulitis score. 0: no infiltration. 1: peri-insulitis. 2: infiltrative insulitis <50% of the islet. 3: infiltrative insulitis >50% of the islet. (**B**) Insulitis score. Female NOD mice were treated with vehicle, 0.1 mg/kg, 0.3 mg/kg or 1 mg/kg CLI-095. Data are collected from 50–60 islets from the pancreas of every mice (three mice in every condition). Every islet was analysed blindly and every islet was given a score between 0 and 3.

We then assessed the impact of TLR4 inhibition on the islet inflammation prior to the onset of diabetes (Fig. 4). To this end, NOD mice were treated with vehicle or different doses of CLI-095 and euthanized at 12 weeks of age. Histological analyses of pancreases revealed that islets were either free of lymphocytes (no insulitis), surrounded (peri-insulitis) or infiltrated by lymphocytes (infiltrative insulitis) (Fig. 4A). Fifty to sixty islets per pancreas were blindly analysed and each islet scored for insulitis: 0 = no insulitis, 1 = peri-insulitis, 2 = infiltration < 50% of the islet and 3 = infiltration > 50% of the islets (Fig. 4A). Control mice (treated with vehicle) and mice treated with the lowest dose of CLI-095 (0.1 μg/ml) had a majority of islets infiltrated with lymphocytes (score 2 and 3) (Fig. 4A). On the other hand, mice treated with the highest doses CLI-095 (0.3 and 1 μg/ml) had a majority of intact islets (score 0) (Fig. 4A). These data indicate that TLR4 blockade decreases the lymphocytic infiltration of the islets.

### TLR4 inhibition prevents the onset of spontaneous diabetes in NOD mice

**Figure 5:**
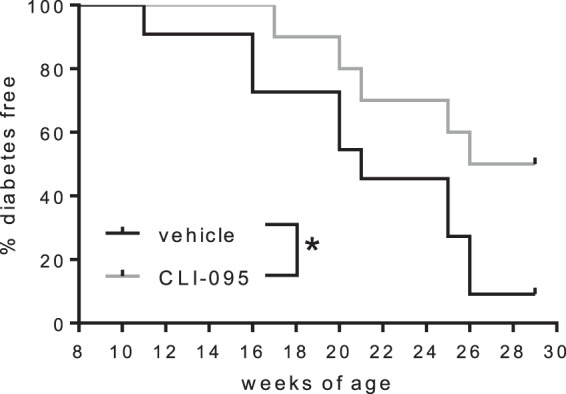
Incidence of spontaneous diabetes in NOD mice. 8-week-old female NOD mice were treated twice a week with 1 mg/ml CLI-095 (red trace, n = 10) or vehicle (black trace, n = 10) and up to 26-weeks of age. Data were collected from 10 mice per group *p ≤ 0.05.

To assess the impact of TLR4 inhibition on the onset of autoimmune diabetes, female NOD mice were treated twice a week with CLI-095 or vehicle, from 8 to 26-weeks of age (Fig. 5). As expected, 90% of control mice (treated with vehicle) developed diabetes by the age of 26 weeks. By contrast, only 50% of mice treated with CLI-095 developed diabetes at that time (Fig. 5). Thus, TLR4 blockade prevents the onset of spontaneous autoimmune diabetes in NOD mice.

## Discussion

We have gathered sufficient evidence suggesting TLR4 is involved in the pathogenesis of diabetes in NOD mice and that its blockade prevents the activation, cellular division, proliferation and differentiation of T lymphocytes into Th1 and Th17 effector lymphocytes. Two studies conducted on TLR4 knock-out mouse models, have previously reported contradictory results on the role of TLR4 in T1D^3,4^. In this study, a pharmacological blockade of TLR4 was used. The TLR4 inhibitor CLI-095 specifically targets the diabetogenic CD4+ T lymphocytes and specifically inhibits TCR-mediated CD4+ T lymphocytes activation. No effect was observed on CD8+ T lymphocytes *in vitro* and *in vivo*. CLI-095 decreases the number of proliferating cells and the number of diabetogenic effector cells in the PLN and in the islets. Treatment with TLR4 inhibitor not only prevents the infiltrative insulitis but also prevents the onset of diabetes. Different mechanisms could explain these results. First, CLI-095 could decrease the inflammation mediated by TLR4 and related to DAMPs^10,11^. This would prevent not only the direct toxicity of DAMPs to β-cells but also the bystander activation of autoreactive T lymphocytes. TLR4 engagement on APC is known to promote a strong Th1 response, which is the main effector mechanism that leads to the destruction of the β-cells^12^. Second, by inhibiting the effect of inflammatory mediators such as CXCL10, TLR4 inhibitor could prevent the recruitment of innate cells and prevent differentiation of lymphocytes into diabetogenic effector cells^13^. In our experiments, TLR4 inhibition decreased specifically the number of IFNγ- and IL-17A-producing CD4+ T cells. Third, by decreasing the inflammation, TLR4 inhibitor could prevent antigen spreading to occur in the islets and therefore decrease the number of autoreactive T lymphocytes^14^. In addition to endogenous ligands and inflammatory mediators that activate TLR4, one has to keep in mind the archetypal TLR4 ligand, LPS, expressed by gram-negative bacteria either from the microbiota or from pathogens. The protective effect of CLI-095 in NOD mice could, in fact, be related to its effect on the gut microbiota. Our research group previously demonstrated that LPS directly induces β-cell death and that TLR4 inhibition preserves viability of β-cells^15^. Moreover, it has been demonstrated that not only diabetic patients, but also patients at risk for T1D have an increased intestinal permeability^16^. This “leaky gut” could facilitate the translocation of microbial components and impact the intestinal immunity^17^. Indeed, it has been observed that bacteria can translocate specifically to the pancreatic lymph nodes and contribute to the pathogenesis of autoimmune diabetes^18^. Recently, it has been shown that β-cells secrete Cathelicidin Related Antimicrobial Peptide (CRAMP) as a protective agent against infiltrating microbes^19^. In addition, CRAMP is also protective against diabetes in NOD mice and increases the number of regulatory dendritic and T cells. Interestingly, CRAMP is defective in female NOD mice, which develop high incidence of autoimmune diabetes. This defect could increase their susceptibility to direct bacterial assault. *Bacteroides dorei*, a gram-negative constituent of the gut microbiota, has been demonstrated to be increased in patients at risk for T1D and in diabetics^20^. *Bacteroides dorei* is usually found in the presence of *Escherichia coli*, in patients at risk^21^. The increased proportion of LPS from Bacteroides in diabetic patients appears to dampen the protective immune response induced by the LPS from *Escherichia coli*. Moreover, injection of LPS from *Bacteroides dorei* in NOD mice is associated with an increased incidence of diabetes^21^. In addition to its capacity to decrease the endotoxin tolerance induce by *Escherichia coli*, *Bacteroides dorei* could directly act on β-cells and thus exacerbate its diabetogenic effect. Regarding the accumulating evidence of the pathogenic effect of *Bacteroides dorei*, TLR4 blockade could still be a preventive treatment in patients at risk for T1D. Lastly, a direct effect of TLR4 ligands on autoreactive T lymphocytes is not excluded, since its expression on CD4+ T cells has been reported^22^.

In summary, further studies are needed to determine the precise mechanism of TLR4 activation in T1D. However, CLI-095 has already undergone a successful phase 1 clinical trial in healthy volunteers, and could potentially be tested clinically^23^. Subsequently, other anti-inflammatory drugs have been tested in humans at risk for T1D with encouraging results. Inhibiting the activation of TLR4, would prevent both inflammatory and autoimmune processes, and thus could be a promising therapeutic approach in T1D^2^.

## Material and Methods

### Animals

NOD/ShiLtJ (NOD), NOD.CgTg(TcraBDC2.5,TcrbBDC2.5)1Doi/DoiJ (BDC2.5), NOD.129S7(B6)-*Rag1*^tm1Mom^/J (NOD/*Rag1*^*−/−*^) were obtained from Charles River (L’Arbresle, France). All animals were maintained under specific pathogen-free conditions and used in accordance with the Guidelines for the Care and Use of Laboratory Animals. Experiments were approved by the Animal experimentation & Animal Welfare Office of the University of Geneva and the Geneva Veterinary Authorities.

### Cell culture medium, reagents and antibodies

Lymphocytes were cultured in RPMI 1640 (Life Technologies, Carlsbad, USA) supplemented with 10% heat-inactivated Fetal Calf Serum (FCS) (Biochrom GmbH, Cambridge, UK), 25 mM HEPES Buffer Solution (Life Technologies, Carlsbad, USA), 1 mM sodium pyruvate solution (Sigma, St-Louis, USA), 0.5 mM 2-mercaptoethanol (Bio-Rad, Hercules, USA), MEM Non-essential Amino Acid Solution 1x (Sigma, St-Louis, USA), 100U/ml Penicillin and 100U/ml Streptomycin (Life Technologies, Carlsbad, USA). Lymphocytes were activated with low endotoxin and azide-free (LEAF) purified anti-mouse CD3ε antibody (145-2C11 or 17A2, Biolegend, San Diego, USA), anti-CD28 (37.51, Milteyni Biotec, Bergisch Gladbach, Germany) and ultrapure Lipopolysaccharide (LPS) from *E. coli 0111:B4* (Invivogen, San Diego, USA), resuspended with endotoxin-free water according to manufacturer’s instructions. When required, cells or mice were also exposed to BDC2.5 mimotope 1040-51 (Lucerna-Chem, Luzern, CH) and TLR4 signalling inhibitor CLI-095 (Invivogen, San Diego, USA) or vehicle (DMSO).

### Splenocyte and lymph nodes cell isolation, culture and activation

Spleens and lymph nodes from mice were harvested and single-cell suspensions were prepared by crushing spleens and lymph nodes through 70 μm cell strainers. Two hundred thousand lymph node cells were rinsed and stained with fluorochrome-conjugated antibodies as described below. Five hundred thousand splenocytes were either stained with fluorochrome-conjugated antibodies as described below or cultured at 37 °C for 24 h with 5 μg/ml anti-CD3 and 1 μg/ml anti-CD28, in presence or absence of 1, 5 and 10 μM of CLI-095 and expression of early activation marker CD69 was evaluated. For antigen-specific activation assay, BDC2.5 splenocytes were stained with 2.5μM CFSE (Life Technologies, Carlsbad, USA) following the manufacturer’s instructions and were stimulated with 0.05, 0.2, 0.5 and 1 μg/ml of BDC2.5 mimotope, in presence or absence of 10μg/ml CLI-095 for 48 h. Proliferation was analysed by CFSE dilution.

### Flow cytometry

Lymphocytes were stained with the following fluorochrome-conjugated antibodies as indicated in the manufacturer’s instructions: FITC anti-mouse CD25 antibody (3C7, Biolegend, San Diego, USA), PE/Cy7 anti-mouse CD69 antibody (H1.2F3, Biolegend, San Diego, USA), PE anti-mouse CD69 antibody (H1.2F3, Biolegend, San Diego, USA), PE/Cy7 anti-mouse CD8a antibody (53-6.7, Biolegend, San Diego, USA), PE anti-mouse CD8a antibody (53-6.7, Biolegend, San Diego, USA), APC/Cy7 anti-mouse CD8a antibody (53-6.7, Biolegend, San Diego, USA), APC anti-mouse CD8a antibody (53-6.7, Biolegend, San Diego, USA), PerCP anti-mouse CD4 antibody (RM4-5, Biolegend, San Diego, USA), PE/Dazzle™ 594 anti-mouse CD4 antibody (GK1.5, Biolegend, San Diego, USA), PE anti-mouse CD3 antibody (17A2, Biolegend, San Diego, USA), PE anti-mouse TCR β chain antibody (H57-597, Biolegend, San Diego, USA), PE anti-mouse CD62L antibody (MEL-14, Biolegend, San Diego, USA), were used. The following isotype control antibodies were used: FITC Rat IgG1 κ isotype Ctrl antibody (RTK2071, Biolegend, San Diego, USA), PE Rat IgG2b κ isotype Ctrl antibody (RTK4530, Biolegend, San Diego, USA), FITC Rat IgG2b κ isotype Ctrl antibody (RTK4530, Biolegend, San Diego, USA), PE Rat IgG2a κ isotype Ctrl antibody (RTK2758, Biolegend, San Diego, USA), PE Armenian Hamster IgG isotype Ctrl antibody (HTK888, Biolegend, San Diego, USA), PE/Cy7 Rat IgG1 κ isotype Ctrl antibody (RTK2071, Biolegend, San Diego, USA).

For intracellular staining cells were stimulated with Cell Activation Cocktail (Biolegend, San Diego, USA) for 4 h, fixed and permeabilized using Intracellular Fixation & Permeabilization Buffer Set (eBioscience, Waltham, USA). Then staining was performed with the following fluorochrome-conjugated antibodies as indicated in the manufacturer’s instructions: FITC anti-mouse IFN-γ antibody (XMG1.2, Biolegend, San Diego, USA), PE/Cy7 anti-mouse IL-17A antibody (TC11-18H10.1, Biolegend, San Diego, USA), PE anti-mouse IL-4 antibody (11B11, Biolegend, San Diego, USA) and PE anti-mouse IL-2 (JES6-5H4, Biolegend, San Diego, USA). For Ki67 staining (BD Pharmingen, Franklin Lakes, USA) cells were fixed and permeabilized using Foxp3 Staining Buffer Set (eBiosciences, Waltham, USA). For both surface staining and intracellular staining Zombie Red™ Fixable Viability Kit (Biolegend) was used to exclude dead cells. Flow cytometry was performed using a Cyan ADP Analyzer (Beckman Coulter). Data were analyzed with FlowJo software (FlowJo, LLC).

### Adoptive transfer model

Accelerated diabetes was induced by adoptive transfer of BDC2.5 CD4+ T lymphocytes, as previously described^24^. Mice were euthanized after 11 days, and spleen and pancreatic lymph nodes were harvested for T lymphocyte analyses as described above.

### Bio-Plex Pro mouse cytokine assay

IL-1β, IL-6, IL-10, IL-17A, IFNγ and TNFα quantifications were performed on sera from vehicle and CLI-095-treated NOD/BDC2.5 mice with Bio-Plex Pro Mouse Cytokine assay (Bio-Rad, Hercules, USA) according to manufacturer’s instructions.

### TLR4 inhibition in NOD mice

Eight-week-old female NOD mice were treated intraperitoneally twice a week with 0.1 mg/ml, 0.3 mg/ml, 1 mg/ml CLI-095 or with vehicle (DMSO). Diabetes was monitored with blood glucose (Precision Xceed Abbott) and mice were considered diabetic when glycaemia was ≥18 mM twice consecutively within 48 hours. Mice were followed until 30-weeks of age.

### Insulitis score

Treated and control NOD mice were euthanized at 12-weeks of age. Pancreases were harvested and fixed in 10% formaldehyde, embedded in paraffin, sectioned and stained with hematoxylin and eosin. Insulitis was evaluated blindly and as previously described^25^. Briefly, 50–60 islets were analysed per pancreas and a score between 0 and 3 was given to each. 0 = no insulitis, 1 = peri-insulitis, 2 = infiltration < 50% of the islet and 3 = infiltration > 50% of the islet.

### Statistical analysis

GraphPad Prism (GraphPad Software, Inc) was used for all statistical analyses. Student *t*-test was used to compare two groups, one-way ANOVA with Turkey’s post-hoc test was used to compare one variable in more than two groups, and two-way ANOVA with Sidak post-hoc test was used to compare 2 variables in more than 2 groups. Diabetes incidence curves were calculated by Kaplan-Meier analysis and differences evaluated by log-rank Mantel-Cox test. Differences between groups were considered significant when p < 0.05. Data are presented as means ± standard error of means (s.e.m).

## Research in Context

### What is already known

- Endogenous ligands and inflammatory mediators involved in autoimmune diabetes have been demonstrated to signal through TLR4
- Diabetic patients and mice have increased intestinal permeability and increased concentration of LPS prior to the diagnosis of diabetes
- Cathelicidin Related Antimicrobial Peptide (CRAMP) are defective in NOD mice

### What is key question?

Is TLR4 involved in the pathogenesis of autoimmune diabetes?

### What are the new findings?

- TLR4 inhibition prevents the onset of autoimmune diabetes
- TLR4 inhibition decreases infiltrative insulitis
- TLR4 inhibition decreases the activation, proliferation and effector CD4+ T lymphocytes

### How might this impact on clinical practice in the foreseeable future?

TLR4 inhibitor, CLI-095, has been successful in phase 1 clinical trial. Subsequently, other anti-inflammatory drugs have been tested in humans at risk for T1D with encouraging results. Inhibiting the activation of TLR4, would prevent both inflammatory and autoimmune processes, and thus could be a promising therapeutic approach in T1D. The immunomodulatory effect of CLI-095 could be part of a preventive strategy that targets patients at risk for type 1 diabetes, but also patients that underwent islet transplantation.
